# Supplementary material for: Hybrid Models and Biological Model Reduction with PyDSTool
Source: PLoS Comput Biol. 2012 Aug 9;8(8):e1002628. doi: 10.1371/journal.pcbi.1002628 (PMC3415397; doi:10.1371/journal.pcbi.1002628)
Supplement: Text S4 — Complete source code for the PyDSTool package (version 0.88.120504). Includes API documentation and help files linking to web pages. This file is identical to the current public release on Sourceforge.net. (ZIP) [file pcbi.1002628.s004.zip › PyDSTool/html/PyDSTool.Toolbox.NineML-pysrc.html]

xml version="1.0" encoding="ascii"?


PyDSTool.Toolbox.NineML


| Home | Trees | Indices | Help | PyDSTool |
| --- | --- | --- | --- | --- |

Package PyDSTool ::
Package Toolbox ::
Module NineML

[hide private]

[frames] | no frames]

# Source Code for Module PyDSTool.Toolbox.NineML

```
  1  """ 
  2  PySCes interface code for systems biology modeling and SBML model markup. 
  3   
  4  This toolbox assumes you have PySCes installed. 
  5   
  6  R. Clewley, 2012 
  7  """ 
  8  from __future__ import division 
  9   
 10  from PyDSTool import * 
 11  from PyDSTool.common import _seq_types, _num_types 
 12  import PyDSTool.Redirector as redirc 
 13   
 14  import numpy as np 
 15  from scipy import linspace, isfinite, sign, alltrue, sometrue 
 16   
 17  import copy, os, sys 
 18   
 19  allODEgens = findGenSubClasses('ODEsystem') 
 20   
 21  # ---------------------------------------------------------------------------- 
 22   
 23  _functions = ['get_nineml_model'] 
 24   
 25  _classes = ['NineMLModel'] 
 26   
 27  _features = [] 
 28   
 29  __all__ = _functions + _classes + _features 
 30   
 31  # ---------------------------------------------------------------------------- 
 32   
 33  try: 
 34      import nineml.abstraction_layer as al 
 35  except ImportError: 
 36      raise ImportError("NineML python API is needed for this toolbox to work") 
 37   
 38   


39 -class NineMLModel(LeafComponent):


40      compatibleGens=allODEgens + ['ExplicitFnGen'] 
 41      targetLangs=targetLangs

 42   
 43   


44 -def get_nineml_model(c, model_name, target='Vode', extra_args=None, 
 45                       alg_args=None, max_t=np.Inf):


46      """component is a NineML abstraction layer component, and 
 47      assumes you have imported a NineML model through the Python API or built it directly. 
 48   
 49      Currently only works for 'flat' models 
 50      """ 
 51      assert c.is_flat(), "Import currently only works for 'flat' models" 
 52   
 53      if alg_args is None: 
 54          alg_args = {} 
 55   
 56      dyn = c.dynamics 
 57   
 58      # lists 
 59      pars = [p.name for p in c.parameters] 
 60      vars = dyn.state_variables_map.keys() 
 61      fns = c.aliases_map.keys() 
 62   
 63      # Turn aliases into function defs: 
 64      # multiple passes until all dependencies resolved 
 65      done = False 
 66      dependencies = {} 
 67      sigs = {} 
 68   
 69      while not done: 
 70          done = all([a.lhs in sigs for a in dyn.aliases]) 
 71          for a in dyn.aliases: 
 72              deps = list(a.rhs_names) 
 73              resolved = True 
 74              fnlist = [] 
 75              sig = [] 
 76              for d in deps: 
 77                  if d in fns: 
 78                      # do we have complete info for this d yet? 
 79                      resolved = resolved and d in sigs 
 80                      fnlist.append(d) 
 81                  elif d in vars: 
 82                      sig.append(d) 
 83              dependencies[a.lhs] = fnlist 
 84              if resolved: 
 85                  for f in fnlist: 
 86                      sig.extend(sigs[f]) 
 87                  sigs[a.lhs] = makeSeqUnique(list(sig)) 
 88   
 89      # Quantity types 
 90      declare_fns = [] 
 91   
 92      for a in dyn.aliases: 
 93          sig = sigs[a.lhs] 
 94          fnspec = QuantSpec(a.lhs, a.rhs) 
 95          # add arguments to the function calls 
 96          for f in dependencies[a.lhs]: 
 97              fi_list = [i for i in range(len(fnspec.parser.tokenized)) if fnspec.parser.tokenized[i] == f] 
 98              offset = 0 
 99              for fi in fi_list: 
100                  arg_spec = QuantSpec('args', '(' + ','.join(sigs[f]) + ')') 
101                  new_defstr = ''.join(fnspec[:fi+1+offset]) + str(arg_spec) + ''.join(fnspec[fi+1+offset:]) 
102                  fnspec = QuantSpec(a.lhs, new_defstr) 
103                  offset += len(arg_spec.parser.tokenized) 
104          declare_fns.append(Fun(str(fnspec), sig, name=a.lhs)) 
105   
106      declare_pars = [Par(p) for p in pars] 
107   
108      targetGen = target + '_ODEsystem' 
109      if target in ['Vode', 'Euler']: 
110          targetlang = 'python' 
111      else: 
112          targetlang = 'c' 
113   
114      reg_MCs = {} 
115      reg_epmaps = {} 
116      reg_info_list = [] 
117      reg_models = {} 
118   
119      all_reg_names = c.regimes_map.keys() 
120      num_regs = len(c.regimes_map) 
121      if num_regs > 1: 
122          is_hybrid = True 
123      else: 
124          is_hybrid = False 
125          # This is the default value, updated to True if there are any 
126          # state assignments for events (even for only one regime) 
127          # r is a singleton regime 
128          r = c.regimes.next() 
129          for e in r.on_conditions: 
130              for s in e.state_assignments: 
131                  is_hybrid = True 
132                  break 
133   
134      if is_hybrid: 
135          vars.append('_regime_') 
136   
137      reg_ix_to_name = {} 
138      for reg_ix, r in enumerate(c.regimes): 
139          reg_ix_to_name[reg_ix] = r.name 
140      reg_name_to_ix = invertMap(reg_ix_to_name) 
141   
142      for reg_ix, r in enumerate(c.regimes): 
143          declare_vars = [] 
144          new_vars = get_regime_model(r, fns, sigs) 
145          if len(new_vars) != len(c.state_variables_map): 
146              if len(new_vars) == 0: 
147                  reg_type = 'ExplicitFn' 
148                  for vname in c.state_variables_map: 
149                      new_vars.append( Var('initcond(%s)'%vname, name=vname, specType='ExpFuncSpec') ) 
150                  if is_hybrid: 
151                      new_vars.append( Var('%i'%reg_ix, name='_regime_', specType='ExpFuncSpec', 
152                                           domain=(int, Discrete, reg_ix)) ) 
153              else: 
154                  # have to make ODEs with 0 for their RHS 
155                  reg_type = targetGen 
156                  for vname in remain(c.state_variables_map, new_vars): 
157                      new_vars.append( Var('0', name=vname, specType='RHSfuncSpec') ) 
158                  if is_hybrid: 
159                      new_vars.append( Var('0', name='_regime_', specType='RHSfuncSpec', 
160                                           domain=(int, Discrete, reg_ix)) ) 
161          else: 
162              reg_type = targetGen 
163              if is_hybrid: 
164                  new_vars.append( Var('0', name='_regime_', specType='RHSfuncSpec', 
165                                       domain=(int, Discrete, reg_ix)) ) 
166          declare_vars.extend(new_vars) 
167          declare_list = declare_pars + declare_fns + declare_vars 
168   
169          reg_spec = NineMLModel(name=r.name) 
170          reg_spec.add(declare_list) 
171   
172          if extra_args is not None: 
173              # filter out the objects with names matching 
174              # those missing in this spec 
175              free = reg_spec.freeSymbols 
176              extra_names = intersect([ea.name for ea in extra_args], free) 
177              for name in extra_names: 
178                  reg_spec.add([ea for ea in extra_args \ 
179                                if ea.name in extra_names]) 
180   
181          reg_spec.flattenSpec(ignoreInputs=True) 
182   
183          # build events for this regime 
184          # event mappings for this regime 
185          epmaps = {} 
186          events = [] 
187          for e in r.on_events: 
188              raise NotImplementedError("Non-transition events not yet implemented") 
189   
190          for e in r.on_conditions: 
191              defq = QuantSpec('rhs', e.trigger.rhs) 
192              toks = defq.parser.tokenized 
193              if '=' in toks: 
194                  ix = toks.index('=') 
195                  dirn = 0 
196              elif '>' in toks: 
197                  ix = toks.index('>') 
198                  dirn = 1 
199              elif '>=' in toks: 
200                  ix = toks.index('>=') 
201                  dirn = 1 
202              elif '<' in toks: 
203                  ix = toks.index('<') 
204                  dirn = -1 
205              elif '<=' in toks: 
206                  ix = toks.index('<=') 
207                  dirn = -1 
208              else: 
209                  raise ValueError("Event type not implemented!") 
210              new_defstr = ''.join(toks[:ix]) + '-' + '(' + ''.join(toks[ix+1:]) + ')' 
211              evnames = [eo.port_name for eo in e.event_outputs] 
212              if evnames == []: 
213                  # no outputs, must create an event name based on LHS of trigger condition 
214                  if "".join(toks[:ix]) == 't': 
215                      evnames = ['time_condition'] 
216                  else: 
217                      # !!! HACKY here, not sure what to do to create a good event name 
218                      evnames = ["".join([tok for tok in toks[:ix] if tok in vars])+'_event'] 
219              if r.name != e.source_regime.name: 
220                  # event doesn't belong with this regime 
221                  continue 
222              else: 
223                  reg_target = e.target_regime.name 
224              for evname in evnames: 
225                  ev_args = {'name': evname, 
226                         'eventtol': 1e-3, 
227                         'eventdelay': 1e-3, 
228                         'starttime': 0, 
229                         'term': is_hybrid 
230                          } 
231                  events.append(Events.makeZeroCrossEvent(new_defstr, 
232                                  dirn, ev_args, targetlang=targetlang, 
233                                  flatspec=reg_spec.flatSpec)) 
234                  edict = {} 
235                  for s in e.state_assignments: 
236                      edict[s.lhs] = s.rhs 
237                  if is_hybrid: 
238                      edict['_regime_'] = str(reg_name_to_ix[reg_target]) 
239                  if len(edict) == 0: 
240                      epmaps[evname] = (evname, reg_target) 
241                  else: 
242                      epmaps[evname] = (evname, (reg_target, 
243                                                 EvMapping(edict, 
244                                                           infodict=dict(pars=pars, 
245                                                                       vars=vars)))) 
246              reg_epmaps[r.name] = epmaps 
247   
248          if reg_type == 'ExplicitFn': 
249              targetGenClassName = 'ExplicitFnGen' 
250          else: 
251              targetGenClassName = targetGen 
252          gen_dict = {'modelspec': reg_spec, 
253                      'target': targetGenClassName, 
254                      'algparams': alg_args} 
255   
256          modelC = ModelConstructor(r.name, 
257                                generatorspecs={reg_spec.name: 
258                                                gen_dict}, 
259                                indepvar=('t',[0,max_t])) 
260          if is_hybrid: 
261              modelC.icvalues = {reg_spec.name: {'_regime_': reg_ix}} 
262   
263          if events != []: 
264              modelC.addEvents(reg_spec.name, events) 
265          reg_model = modelC.getModel() 
266          reg_MCs[r.name] = modelC 
267          reg_models[r.name] = reg_model 
268          reg_MI = intModelInterface(reg_model) 
269   
270          reg_info_list.append(makeModelInfoEntry(reg_MI, all_reg_names, 
271                                        epmaps.values())) 
272   
273      if is_hybrid: 
274          # build MIs, map events, etc. 
275          the_model = Model.HybridModel({'name': model_name, 
276                              'modelInfo': makeModelInfo(reg_info_list)}) 
277      else: 
278          the_model = reg_models.values()[0] 
279      return the_model

280   
281   
282   


283 -def get_regime_model(r, fns, sigs):


284      # Quantity types 
285      declare_vars = [] 
286   
287      # only one regime assumed for now 
288      for dx in r.time_derivatives: 
289          atoms = dx.lhs_atoms 
290          assert 't' in atoms 
291          assert len(atoms) == 2 
292          for a in atoms: 
293              if a != 't': 
294                  vname = a 
295          varspec = QuantSpec('D_'+vname, dx.rhs) 
296          fns_used = intersect(varspec.freeSymbols, fns) 
297          for f in fns_used: 
298              fi = varspec.parser.tokenized.index(f) 
299              new_defstr = ''.join(varspec[:fi+1]) + '(' + ','.join(sigs[f]) + ')' + ''.join(varspec[fi+1:]) 
300              varspec = QuantSpec('D_'+vname, new_defstr) 
301          # no domains known 
302          declare_vars.append(Var(str(varspec), name=vname, specType='RHSfuncSpec')) 
303   
304      return declare_vars

305   
306   
307  ################################################## 
308  #  CODE NOTES FOR FUTURE DEVELOPMENT 
309  ################################################## 
310
```

  


| Home | Trees | Indices | Help | PyDSTool |
| --- | --- | --- | --- | --- |

|  |  |
| --- | --- |
| Generated by Epydoc 3.0.1 on Fri May 4 15:24:19 2012 | http://epydoc.sourceforge.net |
